# Mechanistic insights into the alterations and regulation of the AKT signaling pathway in diabetic retinopathy

**DOI:** 10.1038/s41420-023-01717-2

**Published:** 2023-11-17

**Authors:** Jiayuan Li, Kuangqi Chen, Xiang Li, Xuhong Zhang, Liyue Zhang, Qianjie Yang, Yutong Xia, Chen Xie, Xiawei Wang, Jianping Tong, Ye Shen

**Affiliations:** 1 Department of Ophthalmology, The First Affiliated Hospital of Zhejiang University, Hangzhou, Zhejiang China; 2 Department of Cardiology, The Second Affiliated Hospital of Zhejiang University, Hangzhou, Zhejiang China

**Keywords:** Retinal diseases, Cell biology, Biomarkers

## Abstract

In the early stages of diabetic retinopathy (DR), diabetes-related hyperglycemia directly inhibits the AKT signaling pathway by increasing oxidative stress or inhibiting growth factor expression, which leads to retinal cell apoptosis, nerve proliferation and fundus microvascular disease. However, due to compensatory vascular hyperplasia in the late stage of DR, the vascular endothelial growth factor (VEGF)/phosphatidylinositol 3 kinase (PI3K)/AKT cascade is activated, resulting in opposite levels of AKT regulation compared with the early stage. Studies have shown that many factors, including insulin, insulin-like growth factor-1 (IGF-1), VEGF and others, can regulate the AKT pathway. Disruption of the insulin pathway decreases AKT activation. IGF-1 downregulation decreases the activation of AKT in DR, which abrogates the neuroprotective effect, upregulates VEGF expression and thus induces neovascularization. Although inhibiting VEGF is the main treatment for neovascularization in DR, excessive inhibition may lead to apoptosis in inner retinal neurons. AKT pathway substrates, including mammalian target of rapamycin (mTOR), forkhead box O (FOXO), glycogen synthase kinase-3 (GSK-3)/nuclear factor erythroid 2-related factor 2 (Nrf2), and nuclear factor kappa-B (NF-κB), are a research focus. mTOR inhibitors can delay or prevent retinal microangiopathy, whereas low mTOR activity can decrease retinal protein synthesis. Inactivated AKT fails to inhibit FOXO and thus causes apoptosis. The GSK-3/Nrf2 cascade regulates oxidation and inflammation in DR. NF-κB is activated in diabetic retinas and is involved in inflammation and apoptosis. Many pathways or vital activities, such as the Janus kinase (JAK)/signal transducer and activator of transcription (STAT) and mitogen-activated protein kinase (MAPK) signaling pathways, interact with the AKT pathway to influence DR development. Numerous regulatory methods can simultaneously impact the AKT pathway and other pathways, and it is essential to consider both the connections and interactions between these pathways. In this review, we summarize changes in the AKT signaling pathway in DR and targeted drugs based on these potential sites.

## Facts

- DR is associated with alterations in the AKT pathway, which vary among different retinal cells at different stages of the disease.
- During the progression of DR, the AKT signaling pathway plays an important role in various retinal cells, including retinal neurons, retinal ganglion cells (RGCs), retinal pericytes (RPCs), retinal capillary vascular endothelial cells (RCVECs), and retinal pigment epithelium cells (RPECs), regulating functions such as proliferation, apoptosis, inflammation, angiogenesis, and protein synthesis.
- The regulation of the AKT pathway plays a significant role in the occurrence and progression of DR.

## Open questions

- How do the changes in the levels of AKT and its upstream/downstream molecules at different stages of DR impact the disease progression and the cellular activities involved?
- What is the molecular mechanism of AKT’s role in the process of retinal cytopathic disease in DR, and what are the potential regulatory means and factors?
- Could AKT signaling be a major target for the future treatment of DR?

## Introduction

**Fig. 1:**
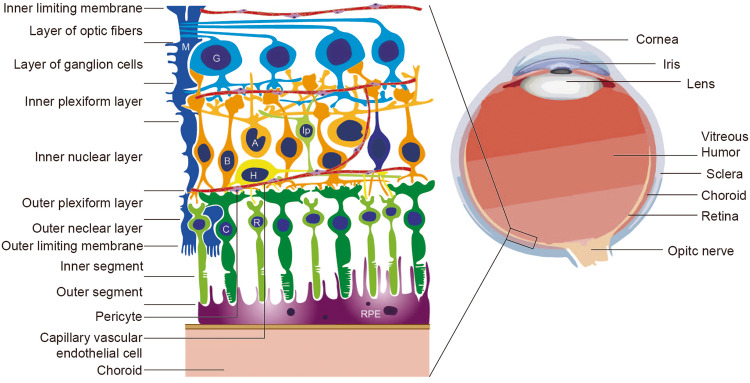
Anatomy of the eye, retina and choroid. Located in the innermost layer of the eye wall, the retina consists of the retinal pigment epithelium (RPE) and nerve layer, which includes rod cells (R), cone cells (C), bipolar cells (B), horizontal cells (H), amacrine cells (A), interplexiform cells (Ip), ganglion cells (G) and Müller cells (M). The choroid is adjacent to the retina and rich in blood vessels that provide oxygen and nutrients to the outer retina. During PDR, neovascularization breaks through the choroid-retina barrier, easily leading to further diseases such as retinal detachment or glaucoma. Vascular endothelial cells and pericytes, as components of capillaries, play an important role in angiogenesis.

Diabetic retinopathy (DR) is the most common and serious ocular complication of diabetes mellitus and damages various structures of the eyeball (Fig. 1). The clinical signs of DR are divided into mild nonproliferative DR (NPDR) and proliferative DR(PDR) [1].

The blood retinal barrier (BRB) is an important structure in the retina consisting with internal components from tight junctions between retinal capillary endothelial cells and external component from tight junctions between retinal pigment epithelium cells (RPECs) [2]. The breakdown of the inner BRB is a hallmark of many degenerative retinal diseases, including DR, and can lead to changes in vascular permeability [3]. In NPDR, large quantities of studies observed BRB damage via retinal pericyte (RPC) loss, retinal capillary vascular endothelial cell (RCVEC) apoptosis and capillary basement membrane thickening. And these symptoms are accompanied by retinal microaneurysm, intraretinal hemorrhage, exudates of abnormal blood vessels and retinal neuron apoptosis. Proliferative diabetic retinopathy (PDR) is characterized by abnormal neovascularization.

The serine/threonine kinase AKT (also known as protein kinase B), which was discovered in 1987, plays an important role in regulating a variety of cellular functions, including metabolism, growth, proliferation, survival, transcription, and protein synthesis, and has become the focus of attention in the medical community [4]. Phosphatidylinositol 3 kinase (PI3K), which was discovered in 1985, is the most important activator of intracellular AKT-mediated signal transduction cascades [5, 6]. Recent discoveries and mechanistic studies on upstream regulators and downstream substrates of the AKT pathway were described in a previous review [7].

Many studies have shown that AKT signaling is attenuated in NPDR. Rats with diabetes induced by a high-fat diet exhibit low levels of AKT and p-AKT in retinal tissues, and this low expression is accompanied by the induction of ROS and decreased phosphorylated mammalian target of rapamycin (mTOR) and nitric oxide synthase, which synthesize NO and affect vascular permeability [8, 9]. Clinical studies on diabetics and nondiabetics examined the changes and the anti-apoptotic effects of AKT in retinal ganglion cells (RGCs) using immunohistochemical techniques, and the expression of the mitochondrial proteins cytochrome c, apoptosis-inducing factor (AIF) and Bad was found to be upregulated in the diabetic retina [10]. Gene Ontology and pathway analyses have shown significant enrichment of the PI3K/AKT signaling pathway in the DR rat retina [11, 12].

However, the AKT pathway is not always inhibited during DR, particularly in RCVECs during neovascularization in PDR. During the early stage of diabetic retinopathy (NPDR), the expression of AKT and p-AKT in the retina exhibited a decrease at 8 and 12 weeks after diabetes induction, but displayed an increase at 4 weeks in a study using streptozotocin-induced diabetic rats [13]. This emphasizes the dynamic changes in AKT signaling during the progression of DR. Additionally, other studies have shown that phosphoenolpyruvate carboxykinase 1 expression is upregulated and AKT is overactivated in the proliferative retinal tissues of PDR patients [14, 15]. The overexpression of phosphoenolpyruvate carboxykinase 1-induced heterotrimeric G proteins promotes vascular endothelial growth factor (VEGF)/VEGFR2-induced endocytosis and downstream activation of AKT/mTOR and mitogen-activated protein kinase (MAPK) [14]. Proteomics analysis of the aqueous humor of PDR patients revealed enrichment of the AKT signaling pathway [16].

Although there is currently no model that can fully mimic the complete pathophysiology of neuronal and vascular changes in both NPDR and PDR, each model recapitulates many of the disease phenotypes [17]. Studies on these DR models have revealed changes and mechanisms of the AKT pathway in DR progression from various aspects. However, the main problem is that there is no retrospective summary of the changes and regulation of AKT and its related factors in the different stages of DR as well as in different retinal cells. Thus, in this review, we summarize the changes and roles of the AKT pathway in DR and review research on select regulatory molecules and drugs that affect the upstream and downstream AKT pathway in DR. We also discuss the crosstalk between the AKT pathway and other pathways involving Janus kinase (JAK)/signal transducer and activator of transcription (STAT) signaling and MAPK signaling.

## AKT-associated retinal destruction and neovascularization in DR

The downregulation of AKT in diabetes leads to cell damages in various cell types, such as cell apoptosis, oxidative stress, and cell cycle impairment in RPCs, cell apoptosis in RCVECs and retinal neurons [18–20]. AKT has been shown to protect these cells from apoptosis by affecting the activity of several transcription factors involved in the regulation of cell survival. However, diabetes and its associated oxidative/inflammatory stress promote an imbalance in the AKT pathway in the retina, leading to significant apoptosis in retinal cells, impaired mitochondrial function, and microvascular degeneration [21–23]. Advanced glycation end product (AGE)-modified substrates, which play important roles in the pathogenesis of diabetes, lead to RPC dysfunction and death by reducing AKT signaling and can induce endothelial to mesenchymal transition [24, 25]. Additionally, AGE receptors are associated with a variety of pathways, such as hypoxia inducible factor-1 (HIF-1), nuclear factor kappa-B (NF-κB), AKT and MAPK, that initiate and maintain an unfavorable proinflammatory state [26]. Moreover, hyperglycemia induces fibronectin, type IV collagen, and laminin expression in human RPECs through the PI3K/AKT signaling pathway, contributing to the formation of fibrotic membranes during DR development [27].

Nonetheless, based on the literature, we hypothesized that AKT is upregulated in RPECs and RCVECs during PDR [16]. The experiment confirmed that treatment with high glucose and AGE increases activation of AKT in cultured human retinal endothelial cells in vitro [28]. Due to hyperlipidemia and microcirculatory disorders, diabetes can cause tissue hypoxia as a result of decreased oxygen carrying and dispersal capacity of blood, as well as metabolic disorders leading to insufficient oxygen utilization [29, 30]. Thus, we hypothesize that retinal hypoxia could induce compensatory vascular hyperplasia, which explains the upregulation of VEGF expression and the activation of the AKT and mTOR pathways in PDR. The induction mechanism of angiogenesis has been examined, and studies have shown that under hypoxic conditions caused by diabetes, RPECs can trigger the HIF-1α pathway to release VEGF and other angiogenic factors [31–33]. The VEGF/VEGFR2-induced PI3K/AKT/endothelial nitric oxide synthase (eNOS) pathway then leads to the production of NO, causing physiological angiogenesis in RCVECs [34]. Certain proteins that promote angiogenesis can be directly or indirectly activated by VEGF/AKT through the intracellular pathway to promote neovascularization [35].

Inhibitors and activators of the PI3K/AKT signaling pathway are utilized in the treatment of diabetic retinopathy. Some commonly used inhibitors include LY294002, wortmannin, and GDC-0941 which suppress the activity of AKT by targeting its upstream molecules [36–38]. On the other hand, activators such as insulin and IGF-1 stimulate the PI3K/AKT pathway leading to a cascade of downstream effects including enhanced glucose uptake and angiogenesis [39, 40]. Additionally, AGE inhibitors such as aminoguanidine are also being studied for their potential ability to modulate the PI3K/AKT pathway in diabetic retinopathy [41].

Further research has uncovered three distinct AKT isoforms: AKT1, AKT2, and AKT3 [42]. While they exhibit an impressive 80% similarity, these homologs display interconnected roles under physiological and pathological conditions, signifying that their functions are not merely redundant. Recent findings suggest that AKT1 and AKT2 mutually regulate each other in RPECs derived from human DR tissues and diabetic mice [43]. A decrease in AKT2 within RPECs triggers a compensatory rise in AKT1, subsequently attenuating DR. Overexpression of AKT1 in RPECs counteracts the retinal abnormalities induced by diabetes, whereas the functional loss of AKT1 in RPECs accelerates retinal vascular damage in diabetic mice [43]. Notably, in RPEC cells from human DR eyes and diabetic mice, there’s an enhanced AKT2 activity, which correlates with epithelial–mesenchymal transition and cellular migration [44]. Moreover, transcriptomic analyses in RPCs reveal a marked reduction in AKT3 levels, playing a pivotal role in RPC functional deficits and BRB alterations [45]. The intricacies of these mechanisms beckon further investigation.

## Upstream regulators of PI3K/AKT signaling in DR

### Insulin

The receptor tyrosine kinase family, which is one of the most important upstream activators of the AKT pathway, includes 20 subfamilies [46]. Among these subfamilies, the insulin receptor family, which is downstream of insulin, can phosphorylate insulin receptor substrate (IRS) and thus activate the PI3K/AKT pathway [47]. Excess hexosamine caused by diabetes blocks the AKT-mediated neuroprotective effect of insulin, inducing apoptosis in retinal neurons [48]. Diabetes progressively impairs the constitutive retinal insulin receptor signaling pathway and decreases the AKT cascade, resulting in various pathologies, such as neuronal cell death and increased vascular permeability [49].

Mechanistically, insulin induces mRNA expression of haem oxygenase-1 (HO-1, an antioxidant and cell protector) through the IRS-1/PI3K/AKT pathway, which has anti-apoptotic properties [50]. The IRS-2 levels are reduced in streptozocin-induced diabetic rats and are responsible for the reduced activity of the retinal IRS/PI3K/AKT pathway [49]. Moreover, researchers compared the results with healthy controls and found that DR mouse RCVECs and RPCs exhibit decreased miR-126 expression, resulting in increased IRS-1 expression and decreased miR-7a cooperation with IRS-2 [51, 52]. Using mimics to induce overexpression inhibits the PI3K/AKT cascade and thereby reduces cell viability and invasion to suppress angiogenesis in PDR.

### IGF-1

Insulin-like growth factor-1 (IGF-1) is a neurotrophin that has been implicated in the pathogenesis of diabetic neurological diseases [53, 54]. In diabetic patients, low expression of IGF-1, particularly in the vitreous humor, has been observed [55]. Low IGF expression may play an important role in the development of new blood vessels in diabetic patients.

Functional studies on RPECs and neuronal cells have shown that PI3K/AKT and MAPK are activated by IGF-1 for cell protection, and blocking the PI3K/AKT pathway eliminates the protective effect of IGF-1, whereas blocking the MAPK pathway is ineffective [56]. In the early stages of DR, the somatostatin levels decline with degenerative changes in photoreceptor cells because somatostatin acts as one of the most important neuroprotective factors in the retina by enhancing IGF-1-mediated AKT phosphorylation [57]. Interestingly, IGF-1 can work synergistically with dopamine to reduce angiogenesis in PDR by downregulating VEGF expression [58].

Studies have shown that therapies targeting the IGF-I signaling pathway can prevent and reverse the development of DR by regulating the AKT pathway. IGF-I treatment can prevent retinal cell death in diabetic rats and potentially help prevent neuronal cell loss in the retina in patients with diabetes [59, 60]. In mouse retinal cells, IGF-1 signaling is upregulated by silencing protein tyrosine phosphatase 1B to prevent nerve degeneration due to its AKT-regulating effect in vitro [61]. The quinic acid derivative KZ-41 functions as a survival factor for RCVECs because its inhibitory effect on caspase-3 activation is dependent on IGF-1/IRS-1/PI3K/AKT signal transduction [62]. Additionally, in RCVECs, treatment with an anti-rat integrin (IL)-associated protein antibody can attenuate aberrant IGF-1 signaling, leading to AKT activation and VEGF synthesis inhibition and thereby preventing or reversing the progression of DR [63].

Studies on IGF-1 at the RNA level offer some insight into potential treatment targets. IGF-1 is a direct target of miR-142-5p and/or miR-18b, and the downregulation of these two microRNAs in the retinal tissues of DR rats and human RCVECs stimulated with hyperglycemia leads to VEGF activation by affecting the IGF-1 and AKT signaling pathways [64, 65].

In addition, IGF-1 binding protein-3 (IGFBP-3) protects the BRB integrity, and this response is independent of IGF-1 and calcium but requires PI3K/AKT activation, suggesting a novel protective effect of IGFBP-3 on the retina [66]. miR-15b and miR-16 can increase the IGFBP-3 levels by reducing the tumor necrosis factor-α (TNF-α) and suppressor of cytokine signaling 3 signaling pathways, which increase RCVEC apoptosis by modulating IRS-1 and protect against apoptosis induced by hyperglycemia in retinal ECs [67, 68]. Therefore, we conclude that the upregulation of IGFBP-3 can have a positive effect on DR and is inextricably linked to the AKT pathway.

### VEGF

Growth factors (mainly VEGF) are upregulated during hypoxia and are important stimulators of retinal neovascularization in PDR [69]. Tissue ischemia or hypoxia can lead to the production of a transcription factor named as hypoxia-inducible factor 1 (HIF-1), which binds to the VEGF gene promoter and initiates the transcription process [70]. The binding of the VEGF receptor to its ligands activates downstream signaling cascades, including the PI3K/AKT, MAPK and other signaling pathways and thereby controls RCVEC survival, proliferation and vascular formation [71].

Inhibiting VEGF and AKT has become one of the main treatments for vascular abnormalities in DR. To date, a range of pharmacological antibodies that target VEGF and prevent neovascularization and PDR progression have been designed [72–75]. New small molecules, such as JP-153 and nanomaterial gold nanoparticles, have been shown to exert therapeutic effects by affecting VEGF to inhibit AKT activation [76, 77]. Herbal or biological extracts, such as erianin, the plant proteolytic enzyme papain, tangeretin, blueberry anthocyanins, plantaginis semen, arctiin, and sanguinarine, can affect VEGF/PI3K/AKT-induced angiogenesis [78–84]. Quercetin, which is an effective polyphenol, can reduce diabetes and diabetic complications. Quercetin can not only inactivate NF-κB signaling by inhibiting MAPK and AKT to inhibit VEGF-induced excessive inflammation and angiogenesis of retinal photoreceptor cells but also increase the level of diabetic retinal neurotrophic factors, thus preventing retinal neurodegeneration caused by oxidation [85–87].

Studies on biomolecules and mechanisms provide new ideas for the treatment of DR through the VEGF/AKT pathway. Some factors or drugs that inhibit angiogenesis and damage to the BRB by inhibiting VEGF/AKT and MAPK include the endothelial growth factor receptor decoy KH902, the IL-linked kinase inhibitor QLT0267, Wnt inhibitory 1, tocotrienol, and ephrinA2/ephrinA1, which regulate vascular development during embryogenesis [88–93]. A similar protective effect of the AKT pathway was achieved by inhibiting N-methyl D-aspartate receptor, β2-glycoprotein I, and erythropoietin expression [94–96]. Aldose reductase inhibition can inhibit the VEGF/PI3K/AKT pathway, which has been demonstrated as an effective solution for addressing the accumulation of sorbitol and fructose in the retina of diabetic animals [97, 98]. The overexpression of the conserved endoplasmic reticulum protein Nogo-B promotes angiogenesis in PDR via the VEGF/PI3K/AKT pathway in an autocrine manner [99]. Silencing Nogo-B improves the integrity of the BRB in DR by increasing the p-AKT level and decreasing the p-ERK1/2 level [100].

VEGF can act as a stimulator for the PI3K/AKT pathway [7], while the expression of VEGF in endothelial cells is mediated by the PI3K/AKT signaling cascade [101]. It’s important to note that although the AKT-mediated pathway is one way of upregulating VEGF expression, it is not the sole mechanism [102, 103]. In recent years, research on microRNAs has developed rapidly. miR-21 and miR-19a can target and activate PTEN, which negatively mediates the PI3K/AKT/VEGF pathway, and inhibiting these two microRNAs can treat DR [104, 105]. Silencing miR-183 and miR-20b-5p can inhibit DR by regulating the VEGF/PI3K/AKT signaling pathway by upregulating BTG1 and inactivating the THBS1 gene, respectively [106, 107]. The upregulation of miR-199a-3p, miR-7, miR-20b and miR-126 can alleviate the increase in cell proliferation, migration and angiogenesis caused by hyperglycemia by reducing VEGF or blocking the VEGF/PI3K/AKT pathway [108–112]. In addition, lncRNA HEIH is highly expressed in the serum of patients with DR and may promote DR by sponging miR-939-targeted VEGF expression and regulating the activation of the PI3K/AKT pathway [113].

Although the current studies on VEGF inhibitors have shown potent antiangiogenic effects, other studies have shown adverse effects of excessive inhibition of this factor, which are related to cell death induced by AKT inhibition [114]. After intravitreal anti-VEGF treatment of PDR, anti-VEGF crunch syndrome may develop, which can lead to sudden blindness in severe cases [115–117]. In addition, due to the suppressive mechanism of VEGF stimulation, AKT phosphorylation, and NO production, low doses of the drug simvastatin are beneficial in preventing pathological neovascularization, whereas high doses are harmful for increasing neovascularization in the retina due to RCVEC apoptosis [118]. Additionally, inhibiting VEGF may cause neuronal apoptosis in the retina [119]. VEGF is a positive regulator of brain-derived neurotrophic factor production in DR that mediates Müller cell viability and neuroprotection by activating AKT signaling [120]. VEGF inhibition significantly increases RGC apoptosis and neuronal apoptosis in the inner nuclear layer in DR [121]. Studies targeting different VEGF family proteins have shown that VEGF-A acts directly on RGCs to promote their survival [122]. VEGF-B inhibits hyperglycemia-induced retinal apoptosis [123]. Therefore, the study of VEGF needs to be more in-depth and comprehensive, and the dose of VEGF inhibitors should be determined carefully.

### Other growth factors

The expression levels of other growth factors are altered due to DR, and these factors can be targets for treatment. The growth differentiation factor 11 (produced in RPECs in retina) treatment of DR is related to the activation of TGF-β/Smad2 and PI3K/AKT/forkhead box O 1 (FOXO1) and the inhibition of the NF-κB pathway to improve pathological changes in mouse RCVECs [124]. Increased levels of hepatocyte growth factor in the retina in diabetes can activate the AKT signaling pathway, prevent RPC apoptosis caused by TNF-α, and protect the BRB [125]. Normal vitreous bodies promote angiogenesis by activating the epidermal growth factor receptor signaling pathway and AKT cascades [126, 127]. Nerve growth factor resists oxidation and inhibits apoptosis by regulating the PI3K/AKT and MAPK signaling pathways, thus protecting RGCs from damage [128]. Stem cell factor can increase NO production in ECs through the PI3K/AKT signaling pathway, suggesting that anti-SCF is a potential treatment [129].

Therefore, given the dual roles of the AKT pathway in angiogenesis and cell survival, the study of VEGF needs to be more in-depth and comprehensive, and the dose should be decided carefully. In vitro or animal experiments should be accompanied by clinical evaluations.

## Substrates of PI3K/AKT signaling in DR

### mTOR

mTOR is an atypical serine/threonine protein kinase that is a member of the PIKK protein family. There are two different complexes (mTORC1 and mTORC2) in the cell, and these complexes can regulate cell signaling pathways by phosphorylating downstream proteins. PI3K/AKT/mTOR is an important signaling pathway associated with oxidative stress-induced DR. Compared to that in muscle and liver, there is a unique set of reactions in the mTORC1 and mTORC2 pathways during diabetes-induced retinal protein conversion: perturbations of mTORC1 rather than mTORC2 in the retina may be associated with reduced protein synthesis under diabetic conditions [130]. Diabetes reduces phosphatidic acid in the retina, leading to decreased mTOR signaling and increased neuronal cell and RPC death [131, 132]. Decreased retinal protein synthesis is associated with decreased mTORC2 activity in DR, but there are no obvious changes in mTORC1 [130].

Therefore, activation of the PI3K/AKT/mTOR signaling pathway can play a protective role. Leukocyte cell-derived chemotaxin 2 (an antiangiogenic factor) increases the level of endothelial tight junction proteins by activating the Tie2/AKT/mTOR signaling pathway and can improve BRB damage associated with diabetes [133]. Curcumin inhibits hyperglycemia-induced inflammatory damage in human RPECs through the ROS-PI3K/AKT/mTOR signaling pathway [134]. Mangiferin and adiponectin treatment inhibits autophagy by promoting activation of the PI3K/AKT/mTOR cascade [135, 136].

However, it is possible that conventional mTOR inhibitors, such as rapamycin, may modulate HIF-1α-mediated downstream activation of growth factors, such as transcriptional regulation of retinal VEGF, to help prevent BRB damage and retinal microangiopathy. These agents are expected to effectively manage disease progression in both NPDR and PDR. In NPDR, mTOR inhibitors inhibit HIF-1α, VEGF, leakage, and disruption of the BRB. These inhibitors inhibit NF-κB as well as downstream inflammatory cytokines, chemokines, and adherent molecules [137]. In PDR, mTOR inhibitors inhibit several growth factors that play key roles in inducing pathological angiogenesis, such as IGF-1, VEGF, etc. [137].

Studies of signaling pathways have revealed the mechanism of the mTOR pathway. The mTOR kinase inhibitor INK128 inhibits the migration of cultured RPCs [138]. The mTORC1 inhibitor REDD1 can play a role in diabetes-induced VEGF expression [139]. Trimetazidine (a metabolic regulator) protects the diabetic retina by inhibiting the PI3K/AKT/mTOR pathway and restoring autophagy in retina [140]. The overexpression of the lncRNA MEG3 inhibits the endothelial to mesenchymal transition by inhibiting PI3K/AKT/mTOR signaling in DR rats and cell models [141]. Finally, miR-7 mimics can reduce the mTOR levels through the PI3K/AKT/mTOR signaling pathway and thereby reduce hyperglycemia-induced damage in RPECs, which indicates that it is a potential therapeutic strategy for the prevention and treatment of DR [142].

In conclusion, the biological processes involving the mTOR pathway are complex and diverse. Its mechanism still needs to be investigated, ad more biological and clinical tests are needed to understand its regulation.

### FOXO

The proapoptotic transcription factor FOXO is a downstream target of AKT. Activated AKT inhibits the function of FOXO by phosphorylating FOXO and promoting cell survival, growth and proliferation [143]. Inhibiting AKT with high glucose activates FOXO, which plays an important role in RPC apoptosis and enhances RCVEC apoptosis and loss [144, 145]. TNF-α and AGEs induce RPC apoptosis by activating the transcription factor FOXO1 [144]. Additionally, inhibiting the upregulation of FOXO6 inhibits hyperglycemia-induced oxidative stress and apoptosis in RPECs, which is mediated by the downregulation of FOXO6 to activate the AKT/nuclear factor erythroid 2-related factor 2 (Nrf2) pathway [146]. Thus, inhibiting FOXO overactivation can prevent and ameliorate DR.

Nonetheless, the transcription factor FOXO plays an important role in vascular hyperplasia in PDR [147]. The activation of FOXO can prevent and improve vascular protection. Epigallocatechin-3 gallate is a polyphenol compound in green tea that inhibits the PI3K/AKT and MAPK pathways, synergistically enhances the antiangiogenic effect of EGCG by activating FOXO transcription factors and is a safe and effective vasoprotective agent for ischemic retinopathy [148–150].

### GSK-3/Nrf2

AKT can phosphorylate glycogen synthase kinase-3 (GSK-3) to keep it inactive and act as an upstream kinase of Nrf2 to promote the expression of Nrf2 [151, 152]. In the early stage of DR, due to the decrease in AKT phosphorylation, the activity of GSK-3 is abnormally increased, and the expression of Nrf2 is inhibited, which is not conducive to the transcription of Nrf2-initiated antioxidant enzymes and the intracellular antioxidant effect [153, 154].

Since GSK-3 is a key kinase associated with neuronal apoptosis in early retinopathy and contributes to endothelial dysfunction and BRB leakage in DR, the use of the GSK-3 inhibitor lithium chloride reduces apoptosis in retinal neurons and protects the retinal integrity [155, 156]. Increasing the survival factor sulfiredoxin-1 and senescence marker protein 30 or inhibiting serine/threonine protein kinase 25 can activate the AKT/GSK-3β/Nrf2 pathway and protect RGCs from hyperglycemia-induced oxidative damage [157–159]. The antioxidant and anti-inflammatory agents pterostilbene, ginsenoside Rg1, sauchinone, lutein, and astaxanthin act on the AKT/GSK-3β/Nrf2 pathway to reduce apoptosis [160–164]. Additionally, as a novel nanomaterial, tetrahedral framework nucleic acids have good biocompatibility and can prevent retinal ischemia‒reperfusion damage caused by oxidative stress by activating the AKT/Nrf2 pathway [165].

### NF-κB

Usually, the NF-κB pathway is activated after the phosphorylation of inhibitory IκB kinase. AKT is the enzyme that phosphorylates lkB kinase. Therefore, AKT is the upstream/regulator controlling NF-κB activation. AKT1 can directly activate NF-κB through a series of intermediate proteins such as IKK (IκB kinase). When AKT1 is activated, it can phosphorylate IKK, leading to the phosphorylation and degradation of IκB, releasing NF-κB to enter the cell nucleus and initiate the transcription of target genes [166, 167]. There is relatively less information about the direct regulation of NF-κB by AKT2 and AKT3, but considering the potential functional overlap among AKT isoforms, they may also be involved in the activation of NF-κB [43]. However, recent studies have shown that AKT2 interacts with AKT1 to exert its effects through the GSK-3/NF-κB axis. However, it has been shown that the events necessary for NF-κB activation (e.g., IκB degradation, nuclear translocation, and increased NF-κB DNA binding) all occur before the increase in AKT phosphorylation [168]. This finding suggests that AKT is a downstream target of NF-κB. Studies have shown that NF-κB is not activated in the normal retina but is activated in the diabetic retina, and the higher the blood glucose and the longer the time, the higher the activity of NF-κB [169]. NF-κB plays an important role in the inflammatory response and participates in the transcriptional regulation of many inflammatory factor genes, such as binding to inflammatory factors and regulating their expression, thereby increasing the speed of apoptosis and new blood vessel proliferation [170].

There have been many studies on the regulation of the NF-κB and AKT pathways. Melatonin is a potent antioxidant that protects various retinal cells from oxidative damage and is an effective activator of AKT in Müller cells, protecting the retina from damage during DR [171]. Melatonin maintains the integrity of the BRB by inhibiting the PI3K/AKT/STAT3/NF-κB signaling pathway and the production of proinflammatory cytokines and proteins, including IL-1β, TNF-α and inducible nitric oxide synthase (iNOS), through the NF-κB pathway [172, 173]. For new targets of retinal disease, anti-CD146 therapy combined with anti-VEGF therapy enhances the damage induced by hypoxia-induced angiogenesis in vitro and in vivo because under hypoxic conditions, CD146 is involved in the activation of the NF-κB, extracellular regulated protein kinase (ERK), and AKT signaling pathways [174]. Preclinical evidence suggests that crocin has cytoprotective, antioxidant, anti-inflammatory, and blood flow-enhancing effects on retinal tissue by activating PI3K/AKT and inhibiting the NF-κB signaling pathway [175, 176]. Additionally, the upregulation of lncRNA SNHG16 promotes diabetes-related human RCVEC dysfunction by activating the NF-κB and PI3K/AKT pathways and thus promoting the proliferation, migration and angiogenesis of RCVECs [177].

## Crosstalk between AKT signaling and other signaling pathways in DR

### JAK/STAT signaling pathway

Apart from the AKT/PI3K pathway, the phosphorylation of JAK2 leads to the initiation of intracellular signaling and the activation of STAT and NF-κB [178]. The JAK/STAT pathway can rapidly transduce signals from the membrane to the nucleus and is widely involved in various physiological and pathological processes, such as cell proliferation, differentiation, apoptosis, inflammation, and tumors [179]. There is a certain activating relationship between the JAK/STAT and AKT pathways, and some drugs exert protective effects through the coregulation of the JAK/STAT and AKT pathways.

Under hyperglycemia stimulation, p-STAT is significantly enhanced, and STAT3 activation increases apoptosis in diabetic RPCs through the TNF-ɑ/AKT/p70S6 kinase signaling pathway [180]. Genipin protects RPEC functional activity, the inflammatory response, and mitochondrial damage by promoting AKT signaling and regulating the expression of the miR-4429/JAK2 signaling axis [181].

In addition, erythropoietin treatment can protect the barrier function of RPECs, induce axonal regeneration and maintain homeostasis in rat retinal neurons. Low-dose erythropoietin has antioxidant effects on organs affected by diabetes and reduces oxidative and nitriding stress in tissues, as well as AGEs in the retina, preventing NPDR microvascular damage in the diabetic retina [182]. These outcomes depend on the activation and joint actions of the JAK2/STAT pathway, MAPK pathway and PI3K/AKT pathway [183–185].

### MAPK signaling pathway

The MAPK signaling pathway is present in most cells in all organisms. This important signal transduction pathway in eukaryotic cells can stimulate the transduction of cell surface signaling to cells and their nuclei and is closely related to cell proliferation, survival, differentiation, apoptosis and other physiological processes [186]. This pathway is composed of three main families: ERKs, Jun kinases and p38MAPKs. Many regulatory factors can affect the MAPK pathway while simultaneously affecting the PI3K/AKT pathway, and the connection and interaction between them cannot be ignored.

Apelin expression is upregulated in RPCs under hyperglycaemic conditions in vitro [187]. Apelin induces proliferation, migration and the expression of cytoskeletal and tightly linked proteins in human RPCs by activating the expression of PI3K/AKT and MAPK signaling pathway proteins, such as AKT and ERK phosphorylation, and promotes retinal vascular permeability in the early stage of DR [188–190]. Systemic injection of an apelin receptor agonist prevents N-methyl-D-aspartate-induced retinal neuronal cell loss [190].

Pituitary adenylate cyclase-activated polypeptide and vasoactive intestinal peptide play beneficial roles in retinal injury associated with diabetic macular edema progression caused by hyperglycaemic injury and can promote neuronal survival in early experimental DR. Their actions are mediated by activation of the PI3K/AKT and mammalian ERK/MAPK kinase signaling pathways [191]. Pituitary adenylate cyclase-activated polypeptide treatment reduces the levels of oxidative stress-induced markers of apoptosis, including HIF-1, multiple heat shock proteins and TNF-α-associated apoptosis-induced ligands; the PI3K/AKT cascade and MAPK cascade are elevated to inhibit apoptosis, resulting in decreased levels of proapoptotic p-p38 MAPK, c-Jun kinases, and activated caspase [192, 193].

Maspin is a potential target protein for the prevention and treatment of PDR. Maspin reduces the mRNA and protein levels of hyperglycemia-induced HIF-1α and VEGF in a dose-dependent manner. In addition, an increase in hyperglycemia-induced p-PI3K and p-AKT is inhibited by maspin [194]. Inhibiting miR-21-5p inhibits hyperglycaemic induction of human retinal EC proliferation and angiogenesis, which may depend in part on the regulation of the PI3K/AKT and MAPK pathways by its target protein maspin [195].

Pigment epithelial-derived factor derivatives induce p-ERK1/2 and p78 to promote p-AKT in Müller glial cells, which are suppressed under diabetic conditions [196]. Pigment epithelial-derived factors inhibit glycosylation-induced retinal vascular permeability [197]. Pigment epithelial-derived factors induce apoptosis by activating PI3K/AKT to downregulate RPC in hyperglycemia [198].

The local administration of glucagon-like peptide-1 (GLP-1) can restore the expression of GLP-1R and reduce the levels of p-AKT and p-ERK1/2 in DR [199]. In NPDR, this peptide can reduce NF-κB, the inflammasome and proinflammatory factors to play an anti-inflammatory role, and it can promote cell survival by increasing anti-apoptotic proteins and activating the GLP-1R/AKT/GSK-3β signaling pathway [200]. Liraglutide (a glucagon-like peptide-1 analog) attenuates AGE-induced RPC migration and retinal neurodegeneration, significantly attenuates the migration of RPCs, and reverses age-induced changes in p-AKT levels [201, 202].

## Conclusion and perspective

**Fig. 2:**
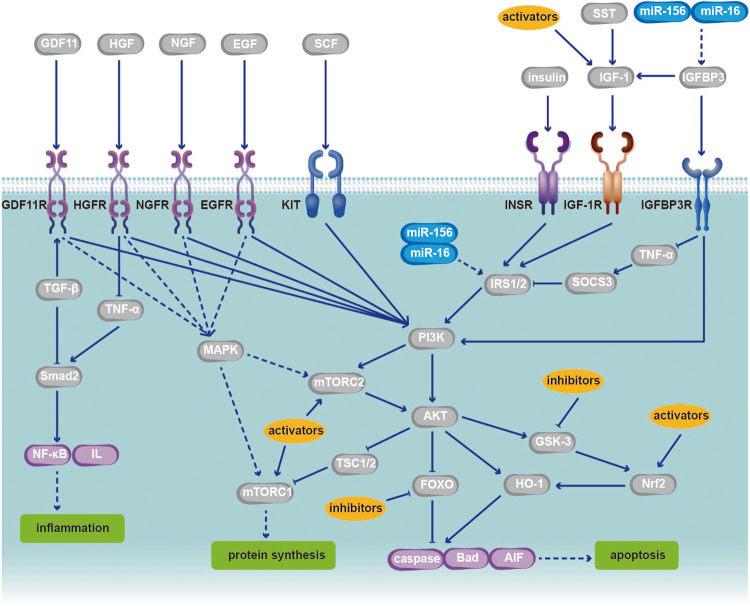
The AKT pathway in the early stage of DR has some important regulatory sites. The early stage of DR, that is, non-PDR, may lead to BRB injury caused by RPC loss, EC apoptosis, capillary basement membrane thickening, retinal microaneurysms, internal retinal bleeding, exudate of abnormal blood vessels, and retinal neuron apoptosis. At this stage, AKT signaling is downregulated due to changes in upstream regulators of AKT signaling, such as insulin and IGF-1, and thus, downstream substrates of AKT exert a concentrated influence on the activity of several transcription factors related to the regulation of cellular protein synthesis, inflammation, apoptosis and other vital activities. Medical or experimental regulation of the disease is mainly aimed at promoting cell survival and reducing cell inflammation and apoptosis, which would result in curbing the retinopathy caused by diabetes. Common and recent activators and inhibitors of different signaling molecular targets in the AKT pathway in the early stage of DR are detailed in Table 2. In addition, many microRNAs also serve as targets for regulating the AKT pathway. GDF11 growth differentiation factor 11, HGF hepatocyte growth factor, EGF epidermal growth factor, NGF nerve growth factor, SCF stem cell factor, TSC1/2 tuberous sclerosis complex 1/2.

**Fig. 3:**
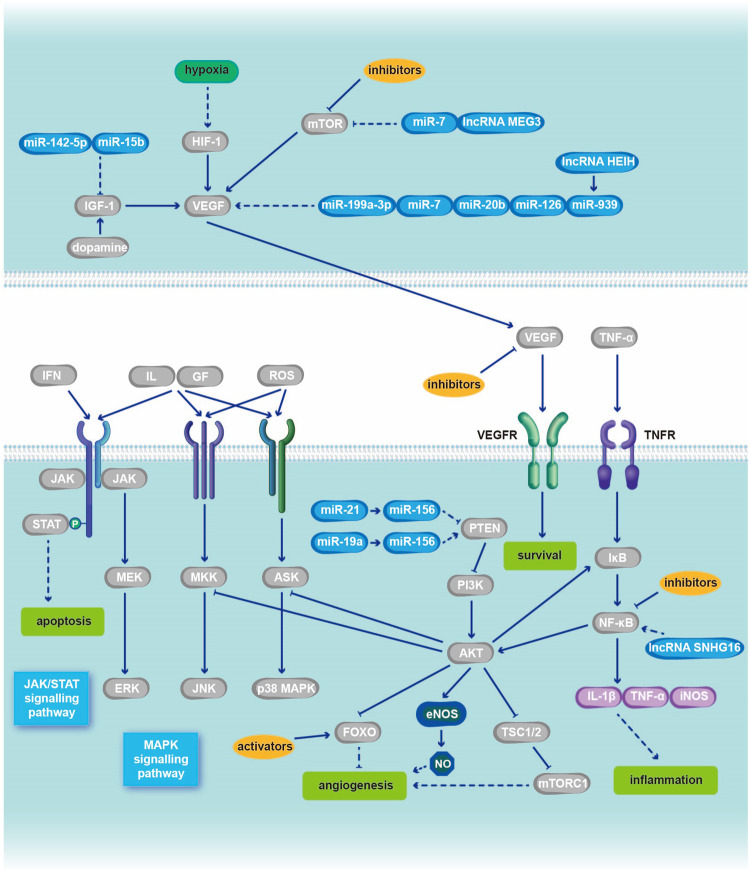
The AKT pathway in PDR has some important regulatory sites. The most significant feature of PDR is pathological neovascularization, which is accompanied by further inflammation and cell apoptosis. Tissue hypoxia or ischemia induces compensatory angiogenesis, which can be regulated by the AKT pathway. At this stage, AKT is upregulated compared to normal levels, contrary to the early stage of DR. Various kinds of antiangiogenic factors are common regulatory methods and research directions for PDR. Among them, VEGF and mTOR are the most frequently used regulatory sites. The research progress relate dot commonly used and latest activators and inhibitors of different signaling molecular targets in the AKT pathway during the PDR period is detailed in Table 2. In addition, many microRNAs also serve as targets for regulating the AKT pathway. However, some antiangiogenic factors, such as VEGF inhibitors, can also lead to increased apoptosis and further deterioration of DR, which should be considered. In addition to the AKT pathway, the interactions between different signaling pathways, such as the JAK/STAT and MAPK pathways discussed in this paper, can also affect each other with the AKT pathway, altering the pathologic process of DR, which suggests that clinical drug selection should not focus on a single pathway. IFN interferon, GF growth factor, ROS reactive oxygen species.

Although many studies on DR have involved signaling pathways, few articles have summarized the signaling pathways related to AKT in the different stages of DR. Our review addresses this unresolved issue by summarizing the manifestations of the AKT pathway in different stages of DR as well as potential targets and therapies related to this pathway (Tables 1 and 2). We found that the AKT pathway is inhibited in early DR and that this inhibition is accompanied by retinal cell apoptosis and structural destruction (Fig. 2). However, during the PDR phase, the AKT signaling pathway is overactivated in retinal cells and retinal capillary ECs (Fig. 3). We suspect that the main reason for this difference in AKT signaling between these two stages is that hypoxia leads to vascular compensatory hyperplasia, which is regulated by a series of signaling factors.

**Table 1:** Classic molecules in AKT signaling in DR.

Molecule name	Molecular function	Subcellular location	Cellular function	Biological function in DR	References
Insulin, INSR	Upstream regulator to activate AKT signaling	Extracellular region	Anti-apoptosis and anti-inflammation	Inhibiting retinal neuron death and avoiding increased vascular permeability	[46, 47, 49]
HO-1	Substrate of AKT and GSK-3/Nrf2	Endoplasmic reticulum membrane	Anti-apoptosis	An antioxidant and cell protective agent	[50, 153]
IRS1/2	The second messenger to activate AKT	Cytoplasm	Anti-apoptosis, and anti-inflammation	Activated by insulin and IGF-1, then activating PI3K/AKT pathway	[47, 49, 50]
IGF-1, IGF-1R	Upstream regulator to activate AKT signaling	Extracellular region	Anti-apoptosis, and anti-angiogenesis	Neuroprotection and inhibiting neovascularization	[53–57]
Somatostatin	Peptide hormones, enhancing IGF-1-mediated AKT phosphorylation	Secreted	Anti-apoptosis	Neuroprotection	[57]
IGFBP-3	Independent upstream regulator of IGF-1 to activate AKT signaling	Secreted	Anti-apoptosis, and anti-angiogenesis	Protecting BRB integrity and stimulating vasodilation	[66–68]
VEGF, VEGFR	Growth factor and its receptor, activating AKT signaling	Extracellular region	Proliferation, anti-apoptosis and angiogenesis	Stimulating retinal neovascularization, mediating cell survival and neuroprotection	[32, 33, 58, 71, 118, 123, 137]
Growth differentiation factor 11	Growth factor, activating AKT signaling	Secreted	Anti-apoptosis and anti-inflammation	Conducive to cell survival by activating TGF-β/Smad2 and PI3K/AKT/FOXO1 pathway and inhibiting NF-κB	[124]
Hepatocyte growth factor	Growth factor, activating AKT signaling	Extracellular region	Anti-apoptosis and anti-inflammation	Preventing RPC apoptosis caused by TNF-α, and protecting the BRB	[125]
Epidermal growth factor	Growth factor, activating AKT signaling	Cell membrane	Anti-apoptosis, anti-inflammation and angiogenesis	Stimulating retinal neovascularization, mediating cell survival	[126, 127]
Nerve growth factor	Growth factor, activating AKT and MAPK signaling	Secreted	Anti-apoptosis and anti-inflammation	Inhibiting RGC apoptosis through AKT and MAPK pathway	[128]
Stem cell factor	Growth factor, activating AKT signaling AKT phosphorylation	Cell membrane, cytoplasm, cytoskeleton	Anti-apoptosis and anti-inflammation	Stimulating NO production through PI3K/AKT pathway as an endothelial permeability factor	[129]
PI3K	Direct regulators of AKT, binding by growth factors	Cytoplasm, cell cortex	Proliferation, anti-apoptosis, anti-inflammation, protein synthesis and angiogenesis	Phosphorylating mTORC2 and AKT	[5, 6, 104, 105, 168, 180]
mTORC2	Direct activating Ser473 of AKT	Cytoplasm	Proliferation, anti-apoptosis, anti-inflammation, protein synthesis and angiogenesis	Phosphorylating AKT	[130]
AKT	Phosphorylating GSK-3, TSC1/2 and FOXO, activating HO-1	Cytoplasm, nucleus	Proliferation, anti-apoptosis, anti-inflammation, protein synthesis and angiogenesis	The core of AKT pathway	[7]
mTORC1	Substrates of AKT	Cytoplasm	Anti-apoptosis, protein synthesis and angiogenesis	Composing endothelial tight junction protein and protecting BRB integrity	[133–136]
FOXO	Substrates of AKT, function ally inhibited by activated AKT	Cytoplasm, nucleus	Anti-proliferation and apoptosis	Enhancing RPC and microvascular apoptosis, and possessing antiangiogenic effect	[143–145]
GSK-3	Substrates of AKT, function ally inhibited by activated AKT	Cytoplasm	Anti-proliferation, apoptosis and anti- angiogenesis	Inducing retinal neuron apoptosis and BRB damage	[151, 152, 155, 156]
Nrf2	Substrates of GSK-3, function ally inhibited by GSK-3	Cytoplasm, cytosol, nucleus	Proliferation, anti-apoptosis	Initiating antioxidant action to reduce cell apoptosis and vascular damage	[153, 154]
NF-κB	Upstream regulator as well as substrates of AKT/IkB signaling	Cytoplasm, nucleus	Apoptosis, inflammation and angiogenesis	Inducing oxidative damage of retinal cells	[168, 170]
IL, IL-1β	Potent proinflammatory cytokine	Lysosome, cytosol	Inflammation	Inducing inflammation and apoptosis	[13, 80, 90]
eNOS, iNOS, NO	Vascular protective and generative factors	Cytoplasm, cell membrane, Golgi apparatus	Protein synthesis and angiogenesis	Inducing retinal ischemia-reperfusion injury and angiogenesis caused by oxidative stress	[8, 34, 89, 118, 173]
TNF-α	Upstream regulator to activate NF-κB	Cell membrane, plasma membrane	Inflammation and apoptosis	Immune defense, inflammation	[125, 173]
Caspase, Bad, AIF	Potent cellar apoptotic factors	Cytoplasm, mitochondrion outer membrane, cytoskeleton	Apoptosis	Inducing apoptosis caused by DNA damage	[10, 50, 62, 192, 193]

**Table 2:** The level changes of main molecules related to AKT pathway in DR and their therapeutic mediation.

Molecule name	Expression level	Patient/Animal models	Cells in retina	Purpose of regulation	Molecules used for regulation	References
AKT	Downregulated in the early stage of DR	Patient, rats, mouse, monkeys, cows, cells in vitro	Retinal neurons, RGCs, RPCs, RCVECs, RPECs	To increase its expression level or activate it	Insulin [39]; IGF-1 [40]	[8–10]
	Upregulated in PDR	Patient, rats, mouse	RCVECs, RPECs	To decrease its expression level or inhibit it	LY294002 [36]; wortmannin [37]; GDC-0941 [38]	[13–16]
Insulin	Downregulated	Patient	Retinal neurons, RPCs, RCVECs	To increase its expression level or activate it	/	[48]
IRS	Downregulated	Rats, mouse	RCVECs, RPECs	To increase its expression level or activate it	miR-126 [51]; miR-7a [52]	[49]
IGF-1	Downregulated	Patient, rats, mouse	Retinal neurons, RGCs, RPCs, RCVECs, RPECs	To increase its expression level or activate it	IGF-1 [59, 60], protein tyrosine phosphatase 1B [61]; quinic acid derivative KZ-41 [62]; anti-rat integrin-associated protein antibody [63]; miR-142-5p [64]; miR-18b [65]	[56]
IGFBP-3	Downregulated	Patient, mouse	RCVECs	To increase its expression level or activate it	miR-15b, miR-16 [67, 68]	[63]
VEGF	Upregulated	Patient, rats, mouse	RCVECs, RPECs	To decrease its expression level or inhibit it	IGF-1 and dopamine [58]; traditional VEGF inhibitors [72–75]; new small molecules or biological factors [76, 77, 88–100]; herbal or biological extracts [78–84]; quercetin [85–87]; miR-21 [104]; miR-19a [105]; miR-183 [106]; miRNA-20b-5p [107]; miR-199a-3p [108]; miR-7 [109]; miR-20b [110]; miR-126 [111, 112]; lncRNA HEIH, miR-939 [113]	[71]
	Excessive inhibition in medical procedures	Patient	Retinal neurons, RGCs	To reduce negative effects of anti-VEGF therapy	/	[118, 120, 121]
mTOR	Downregulated in the early stage of DR	Patient, rats, cells in vitro	Retinal neurons, RPCs, RPECs	To increase its expression level or activate it	Leukocyte cell-derived chemotaxin 2 [133]; curcumin [134]; mangiferin [135]; adiponectin [136]	[130, 131]
	Upregulated in PDR	Patient, pig, mouse	RCVECs, RPECs	To decrease its expression level or inhibit it	Conventional mTOR inhibitors, such as rapamycin [137]; INK128 [138]; REDD1 [139]; trimetazidine [140]; lncRNA MEG3 [141]; miR-7 [142]	[14]
FOXO	Upregulated	Patient	RPCs, RCVECs, RPECs	To decrease its expression level or inhibit it	/	[143–145]
	Activation in medical procedures to suppresses angiogenesis	Cells in vitro	RCVECs	To increase its expression level or activate it	Epigallocatechin-3 gallate [148, 150]	[147]
GSK-3	Upregulated	Patient, cells in vitro	Retinal neurons, RGCs, RPCs, RCVECs, RPECs	To decrease its expression level or inhibit it	Lithium chloride [155, 156]; pterostilbene [160]; ginsenoside Rg1 [161]; sauchinone [162]	[154]
Nrf2	Downregulated	Patient, cells in vitro	Retinal neurons, RGCs, RPCs, RCVECs, RPECs	To increase its expression or activate it	Lutein [163]; astaxanthin [164]; tetrahedral frame nucleic acids [165]	[153]
NF-κB	Upregulated	Patient, rats, cells in vitro	Retinal neurons, RGCs, RPCs, RCVECs, RPECs	To decrease its expression level or inhibit it	Melatonin [171–173]; anti-CD146 [174]; crocin [175, 176]; lncRNA SNHG16 [177]	[168, 170] (205)

Methods for regulating the AKT pathway, including its activation and inhibition, such as routine insulin therapy, IGF-1 therapy and the very popular growth factor therapy (particularly VEGF), have received considerable attention. An increasing number of potential targets, drugs and bioderived health products have been gradually discovered and used in clinical research. Furthermore, substrate studies targeting the AKT pathway provide other ideas for the treatment of AKT-related DR.

However, there remain many limitations to the study of signaling factors, which may become the directions of future research. For example, the response of PDR patients to VEGF treatment is heterogeneous, suggesting that the pathological basis of this condition is multifactorial in nature [203]. The application of VEGF inhibitors clearly needs more clinical evaluation. A similar situation exists with mTOR [204].

The interaction between different signaling pathways in organisms should not be ignored, although the study of these interactions is far from sufficient. Crosstalk was proposed in the 1980s to explain the unlikely pharmacological effects of drugs [204]. Due to the complex and delicate interactions between signaling pathways in organisms, the study of signaling pathways and drug development faces great challenges.

Overall, we are aware that despite many research achievements on AKT-related DR, more in-depth and comprehensive studies on the pathway mechanism are still lacking due to the complexity of the related signaling pathways. More effective drugs targeting AKT signaling are still needed to treat DR at the source, and more clinical evaluation should be conducted.

## Data Availability

We searched the PubMed database (https://pubmed.ncbi.nlm.nih.gov/) using the keywords “diabetic retinopathy AND AKT”, and mainly focused on the recent studies and reviews (published between 2002 and 2022). Additional studies were discovered by consulting the reference lists of the selected articles. Our figures were edited with Adobe Illustrator CC 2018 software (Adobe, San Jose, CA).
